# Noradrenaline-induced release of newly-synthesized accumbal dopamine: differential role of alpha- and beta-adrenoceptors

**DOI:** 10.3389/fncel.2014.00243

**Published:** 2014-08-21

**Authors:** Francisca Meyer, Judith Latour, Alexander R. Cools, Michel M. M. Verheij

**Affiliations:** ^1^Department of Molecular Animal Physiology, Donders Institute for Brain, Cognition and Behaviour, Nijmegen Center for Molecular Life Sciences, Radboud University NijmegenNijmegen, Netherlands; ^2^Department of Cognitive Neuroscience, Donders Institute for Brain, Cognition and Behaviour, Radboud University Nijmegen Medical CentreNijmegen, Netherlands

**Keywords:** noradrenaline-dopamine interactions, norepinephrine-dopamine interactions, alpha-adrenoceptors, beta-adrenoceptors, phentolamine, isoproterenol, alpha-methyl-para-tyrosine, nucleus accumbens

## Abstract

Previous studies have shown that intra-accumbens infusion of isoproterenol (ISO), a beta-adrenoceptor-agonist, and phenylephrine (PE), an alpha-adrenoceptor-agonist, increase the release of accumbal dopamine (DA). In the present study we analyzed whether the ISO-induced release of DA is sensitive to pretreatment with the DA synthesis inhibitor alpha-methyl-para-tyrosine (AMPT). Earlier studies have shown that the PE-induced release of DA is derived from DA pools that are resistant to AMPT. In addition to PE, the alpha-adrenoceptor-antagonist phentolamine (PA) was also found to increase accumbal DA release. Therefore, we investigated whether similar to the DA-increasing effect of PE, the DA increase induced by PA is resistant to AMPT. Pretreatment with AMPT prevented the ISO-induced increase of accumbal DA. The accumbal DA increase after PA was not reduced by the DA synthesis inhibitor, independently of the amount of DA released. These results show that mesolimbic beta-, but not alpha-adrenoceptors, control the release of accumbal newly-synthesized DA pools. The DA-increasing effects of PE have previously been ascribed to stimulation of presynaptic receptors located on noradrenergic terminals, whereas the DA-increasing effects of PA and ISO have been ascribed to an action of these drugs at postsynaptic receptors on dopaminergic terminals. The fact that AMPT did not affect the accumbal DA response to PE and PA, whereas it did prevent the accumbal DA increase to ISO, supports our previously reported hypothesis that the noradrenergic neurons of the nucleus accumbens containing presynaptic alpha-adrenoceptors impinge upon the dopaminergic terminals in the nucleus accumbens containing postsynaptic adrenoceptors of the alpha but not of the beta type. The putative therapeutic effects of noradrenergic agents in the treatment of DA-related disorders are shortly discussed.

## Introduction

It has previously been demonstrated that the release of mesolimbic noradrenaline directs the release of mesolimbic dopamine (DA) (*in vitro* studies: Nurse et al., 1984; Russell et al., 1993; *in vivo* studies: Cools and Tuinstra, 2003; Verheij and Cools, 2008). Both, stimulation of accumbal beta-adrenoceptors by the agonist isoproterenol (ISO), and inhibition of accumbal alpha-adrenoceptors by the antagonist phentolamine (PA) have been found to facilitate accumbal DA release (see Figure 1 in Tuinstra and Cools, 2000; Verheij and Cools, 2009b). The DA increase induced by ISO and PA have previously been ascribed to the binding of these agents at accumbal postsynaptic adrenoceptors located on dopaminergic nerve terminals (see Figure 1 in Tuinstra and Cools, 2000; Verheij and Cools, 2009b). The mainly postsynaptic action of these agents has been confirmed in more recent studies showing that intra-accumbal administration of various DA-increasing doses of either beta-adrenoceptor agonists or alpha-adrenoceptor antagonists (Tuinstra and Cools, 2000; Aono et al., 2013), did not affect accumbal noradrenaline levels (Aono et al., 2007, 2013).

**Figure 1:**
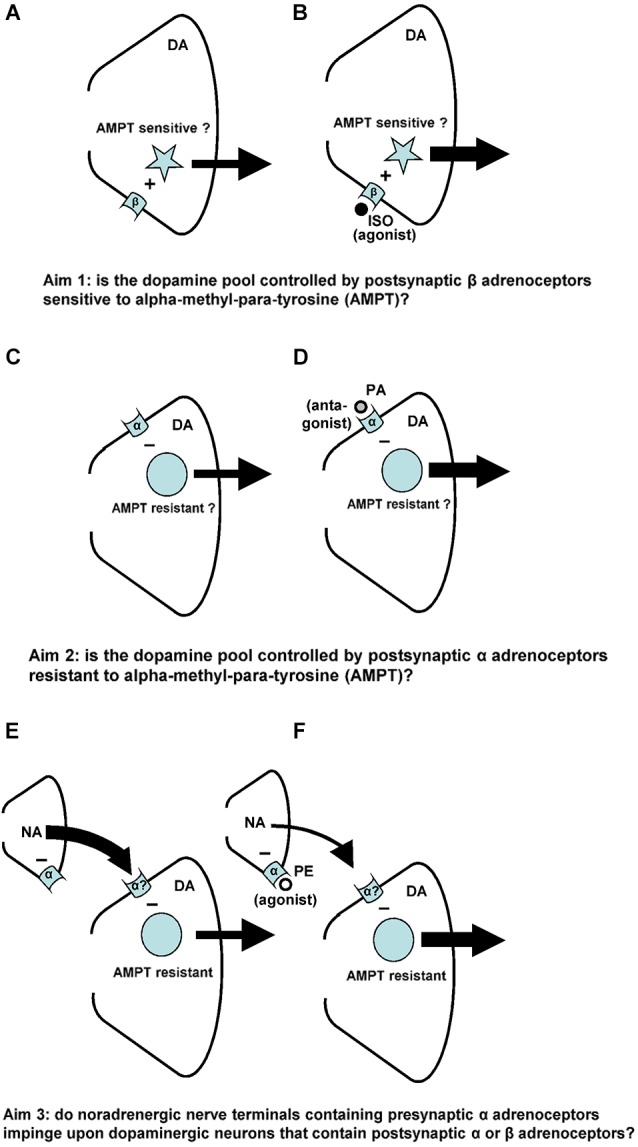
**Noradrenaline-(NA)-induced changes in accumbal DA release**. Postsynaptic beta-(β)-adrenoceptors stimulate (+) the release of accumbal DA **(A)**, whereas postsynaptic alpha-(α)-adrenoceptors inhibit (−) the release of accumbal DA **(C)**. The first aim of the study was to investigate whether the postsynaptic acting beta-adrenoceptor agonist ISO increases DA release from AMPT sensitive pools of DA **(B)**. The second aim of the study was to investigate whether the postsynaptic acting alpha-adrenoceptor antagonist PA increases DA release from AMPT-resistant pools of DA **(D)**. Presynaptic alpha-(α)-adrenoceptors regulate accumbal NA levels **(E)**. The presynaptic acting alpha-adrenoceptor agonist PE is known to reduce accumbal NA release **(F)**. The third aim of this study was to investigate whether the noradrenergic neurons that contain presynaptic alpha-adrenoceptors impinge upon dopaminergic neurons that contain postsynaptic alpha-, but not beta-adrenoceptors **(F)**.

Interestingly, phenylephrine (PE), an alpha-adrenoceptor agonist, has been found to act mainly at the presynaptic adrenoceptors of the nucleus accumbens (see Figure 1 in Tuinstra and Cools, 2000; Verheij and Cools, 2009a). As expected, stimulation of these presynaptic receptors by PE leads to a decreased accumbal noradrenaline release (see Figure 1 in Aono et al., 2007) that in turn was found to result in an increase of the extracellular levels of accumbal DA (see Figure 1 in Tuinstra and Cools, 2000; Verheij and Cools, 2009a). Given that a decrease of accumbal noradrenaline at the level of the DA-stimulating postsynaptic beta-adrenoceptors would have resulted in a reduction of the release of accumbal DA (see also: Mizoguchi et al., 2008), it has previously been hypothesized that those noradrenergic neurons that contain presynaptic alpha-adrenoceptors do not impinge upon dopaminergic terminals that contain postsynaptic adrenoceptors of the beta type (see Figure 1 in Tuinstra and Cools, 2000). Accordingly, the finding that PE increased accumbal DA release suggests that those noradrenergic neurons that contain presynaptic alpha-adrenoceptors, impinge upon dopaminergic terminals that contain postsynaptic adrenoceptors of the DA-inhibitory alpha type (see Figure 1).

Accumbal DA can be released from two types of DA pools (Ewing et al., 1983; Leviel et al., 1989; Verheij and Cools, 2007, 2009b). The first pool consists of newly-synthesized DA and is sensitive to the DA synthesis inhibitor AMPT, whereas the second pool consists of stored DA and is sensitive to the DA vesicle depleter reserpine (RES; for review: Verheij and Cools, 2008). Microdialysis studies have shown that RES counteracts the PA-, but not the ISO-induced accumbal DA release (Verheij and Cools, 2009b). The first aim of the present study was to investigate whether AMPT may counteract the ISO-induced increase of accumbal DA release (see Figures 1A,B). We have previously shown that the PE-induced release of DA is derived from DA pools that are insensitive to AMPT (Verheij and Cools, 2009a). The second aim of the present study was to investigate whether the accumbal DA increasing effects of the postsynaptic acting alpha-agent PA are, in addition to the accumbal DA increasing effects of the presynaptic acting alpha-agent PE, resistant to AMPT (see Figures 1C,D). The third and final aim of the present study was to provide additional evidence for the abovementioned hypothesis that the presynaptic alpha-adrenoceptor of the nucleus accumbens are located on those noradrenergic nerve terminals that impinge upon dopaminergic neurons containing postsynaptic alpha-, but not beta-adrenoceptors (see Figures 1E,F).

The subjects used in the present study were low responder (LR) and high responder (HR) to novelty rats (Piazza et al., 1989; Dellu et al., 1996; Bevins et al., 1997; Cools and Gingras, 1998; Cools and Tuinstra, 2003; Kabbaj, 2004; Verheij et al., 2009; Verheij and Cools, 2011). Given that intra-accumbens administration of PA has previously been found to result in a larger release of accumbal DA in LR than in HR (Tuinstra and Cools, 2000), these rats represent a useful tool to study whether the expected lack of effects of AMPT in PA-treated rats is (in)dependent on the amount of DA that is released.

## Materials and methods

### Subjects

Adult male LR (*n* = 51) and HR (*n* = 51) rats of 180–220 g were selected from the outbred strain of Nijmegen Wistar rats (see Section Open-Field Selection Procedure). All rats were reared and housed in Macrolon cages (42 × 26 × 15 cm; *n* = 3–4 per cage) under a fixed 12/12 h light/dark cycle (lights on: 07.00 a.m.) in a temperature-controlled room (21 ± 1.7°C). Water and food pellets (Ssniff, Soest, Germany) were available ad libitum, except during the testing periods. All experiments were performed in accordance with institutional, national and international guidelines for animal care and welfare (see NRC 2003 guidelines). Every effort was made to minimize the number of animals used and their suffering.

### Selection of LR and HR to novelty

Rats were individually housed 3 days before the open-field selection procedure (Tuinstra and Cools, 2000; Verheij and Cools, 2007). Testing took place between 09.00 h and 17.00 h in a room illuminated by white light of 170 Lux at the middle of the open-field. Rats were placed on a black square table with no walls (160 × 160 cm) for a period of 30 min. This open-field was 95 cm elevated above the floor and surrounded by a white neutral background (270 × 270 × 270 cm). As described by Cools et al. (1990), behavior was recorded with a computerized tracking system. Both ambulation and habituation time were used to select LR and HR. Ambulation was defined as the overall distance (cm) traveled in 30 min. Habituation time was defined as the duration of the period(s) that started as soon as the rat began to explore the open-field and ended as soon as the locomotor activity stopped for at least 90 s. Rats that habituated in less than 480 s and walked less than 4800 cm in 30 min were labeled LR, whereas rats that habituated after 840 s and walked more than 6000 cm in 30 min were labeled HR (Tuinstra and Cools, 2000; Verheij and Cools, 2007). Habituation time in addition to ambulation was used as selection criterion, because traveled distance *per se* is not always a reliable criterion (Cools et al., 1997; Saigusa et al., 1999). Rats that did not fulfill the criteria were excluded from this study. Efforts were made to use these rats in other studies.

### Surgery

One day after the open-field selection procedure took place, LR and HR were unilaterally implanted with a stainless steel guide cannula (length: 5.5 mm, outer diameter: 0.65 mm, inner diameter: 0.3 mm) directed to the right nucleus accumbens according to previously described procedures (Tuinstra and Cools, 2000; Verheij and Cools, 2007). Rats, anesthetized with pentobarbital (60 mg/kg, i.p.), were placed in a stereotactic apparatus and the following coordinates were used according to the atlas of Paxinos and Watson (1986): anterior: +10.6 mm (relative to the interaural line) and lateral: −1.5 mm (relative to the midline suture). The guide cannula was lowered 5.5 mm relative to the dura surface resulting in a vertical coordinate of +3.5 mm for the cannula tip. Finally, the cannula was angled 10° laterally to the right side. The rats were allowed to recover from surgery for the next 7–10 days in Plexiglas dialysis cages (25 × 25 × 35 cm) covered with sawdust on the floor. On 3 consecutive days just prior to the start of the microdialysis experiment, each rat was gently picked up in order to habituate to the procedure assessed on the day when the concentration of accumbal DA was measured. This handling procedure was repeated three times per day.

### Microdialysis

As previously described, a dialysis probe (type A-I-8-02, outer diameter: 0.22 mm, 50,000-molecular-weight cut-off, Eicom, Tokyo, Japan) was carefully inserted into the brain of a conscious rat and secured to the guide cannula with a screw (Tuinstra and Cools, 2000; Verheij and Cools, 2007). The tip of the dialysis probe protruded 2 mm below the distal end of the guide cannula into the nucleus accumbens. The probes had an *in vitro* recovery of 10–12% for DA. The inlet and outlet of the probe were connected to a swivel allowing the rat to move undisturbed. Accumbal dialysates were analyzed for DA (pg/40 μl) according to previously described procedures (Tuinstra and Cools, 2000; Verheij and Cools, 2007). Briefly, the probe was perfused at a rate of 2.0 μl/min with modified Ringer solution (see Section Compounds) and the outflow was collected in a sample loop and injected, once every 20 min, into a high performance liquid chromatography (HPLC) system. DA was separated from the remaining neurotransmitters by means of reversed phase, ion-paring, liquid chromatography and the concentration was measured using electrochemical detection (ECD). The HPLC-ECD system (HTEC-500: Eicom, Tokyo, Japan) was calibrated with a standard DA solution before and after each experiment. The detection limit was 500 fg per sample.

### Effects of phentolamine and isoproterenol in rats treated with alpha-methyl-para-tyrosine

At 4 h following probe insertion, the extracellular accumbal concentration of DA is known to reach a stable baseline ±10% (Saigusa et al., 1999; Tuinstra and Cools, 2000; De Leonibus et al., 2006; Verheij and Cools, 2007; Verheij et al., 2008). As soon as a stable baseline concentration of DA was reached, rats were treated with AMPT according to previously described procedures (Saigusa et al., 1999). In short, 0.1 mM of AMPT or its solvent was locally infused into the nucleus accumbens (40 min: 2 μl/min) whereafter the rats were immediately exposed to a novel cage (Saigusa et al., 1999; Verheij and Cools, 2011). This novel cage was slightly larger than the home cage (new dimensions: 30 × 30 × 35 cm) and lacked sawdust on the floor (Saigusa et al., 1999). It has previously been shown that after open-field selection, HR and LR rats still differ in locomotor activity when exposed to this new cage (see Saigusa et al., 1999; Verheij and Cools, 2011). Rats were exposed to the novel cage, because this environmental challenge strongly facilitates the AMPT-induced DA decrease (Saigusa et al., 1999; Verheij and Cools, 2007). At 100 min after AMPT or its solvent (see also Section Compounds), rats were subjected to a 40-min-lasting intra-accumbens infusion of 0.001 mM of the beta-adrenoceptor-agonist ISO (solvent AMPT: LR: *n* = 8, HR: *n* = 8; AMPT: LR: *n* = 8, HR: *n* = 8) or 0.01 mM (2 μl/min) or the alpha-adrenoceptor-antagonist PA (solvent AMPT: LR: *n* = 8, HR: *n* = 8; AMPT: LR: *n* = 9, HR: *n* = 10). Infusion of modified Ringer solution (2 μl/min, 40 min) served as control for PA and ISO treatment (solvent AMPT: LR: *n* = 9, HR: *n* = 9, AMPT: LR: *n* = 8, HR: *n* = 8). All rats were randomly divided over the different treatment groups.

The noradrenergic drugs were administered 100 min after AMPT, because at this time AMPT still reduced the levels of accumbal DA (see Section Results and Watanabe et al., 2005), whereas the amount of accumbal noradrenaline has previously been found to be returned to baseline levels (Verheij and Cools, 2009a). The dose of PA and ISO were chosen because these doses have been shown to increase extracellular levels of DA in the nucleus accumbens in a receptor-specific manner (Tuinstra and Cools, 2000; Verheij and Cools, 2009b).

### Histology

At the end of each experiment the rat was deeply anesthetized with an overdose of sodium-pentobarbital (60 mg, i.p.) and intracardially perfused with 60 ml 4% paraformaldehyde solution. Vibratome sections (100 μm, Leica VT1000F; Leica, Rijswijk, The Netherlands) were cut to determine the exact location of the microdialysis probe.

### Compounds

The following compounds and solutions were used (Tuinstra and Cools, 2000; Verheij and Cools, 2009b): (1) ISO-hydrochloride and PA-hydrochloride (Sigma, St Louis, USA); (2) dl-AMPT-hydrochloride (Axel Kistner AB Fack, Göteborg, Sweden); and (3) modified Ringer solution: 147 mM NaCl, 4 mM KCl, 1.1 mM CaCl2.2H2O and 1.1 mM MgCl2.6H2O were dissolved in ultra pure water (pH 6.0). The Ringer solution served as solvent for all compounds.

### Statistical analysis

The drug-induced changes of accumbal DA were expressed as a percentage of the concentration of DA that was measured in the 20-min-lasting period just before these drugs were infused. All data are expressed as the mean ± SEM. A three-way ANOVA with the factors rat type, treatment, and time (for repeated measures) was assessed followed by a *post-hoc* Student’s *t*-test where appropriate. A probability level of *p* < 0.05 was taken as significant. SPSS for Windows (Release 12) was used to statistically analyze the data.

## Results

### Open-field selection procedure

The open-field selection procedure revealed 25% LR and 26% HR. The average distance traveled in 30 min (±SEM) was 3425 ± 123.6 cm and 8273 ± 233.0 cm in LR and HR, respectively. The average habituation time (±SEM) was 353 ± 20.8 s in LR and 1322 ± 58.2 s in HR.

### Histology

**Figure 2:**
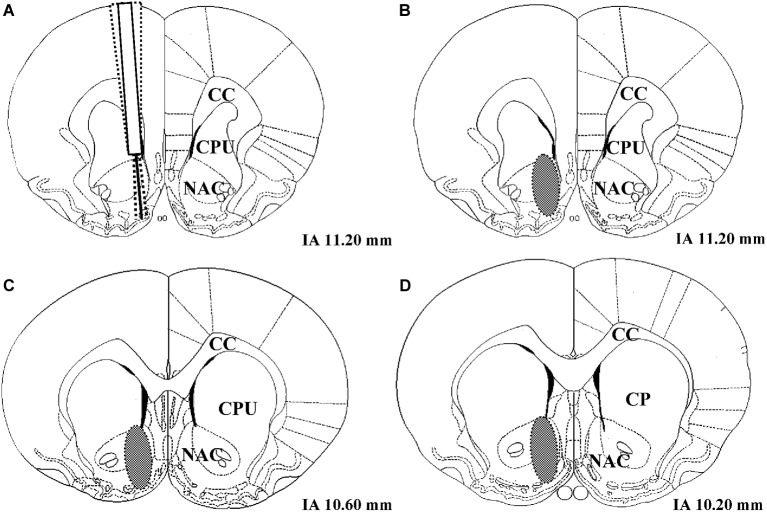
**(A)** Example of three unilateral microdialysis probe tracks located in the right nucleus accumbens. The probe protrudes 2 mm below the distal end of the guide cannula. **(B–D)** Schematic illustration of coronal brain sections containing the nucleus accumbens. The brain region in which correctly placed probes were found is indicated as a gray oval. IA corresponds to the distance (mm) from the interaural line according to Paxinos and Watson (1986), NAC = nucleus accumbens, CPU = caudate putamen, CC = corpus callosum.

Following histological verification, 1 LR treated with the solvent of AMPT and PA had to be excluded from the analysis due to an incorrect placement of the microdialysis probe (number of rats included in data analysis: 51 − 1 = 50 LR and 51 − 0 = 51 HR). The coronal region of the nucleus accumbens in which all correctly placed microdialysis probe tracks were located is shown in Figure 2.

### Basal levels of dopamine

Baseline extracellular levels of accumbal DA were 3.8 ± 0.15 pg/sample in LR and 4.40 pg ± 0.31 pg/sample in HR (rat type effect: *F*_(1,99)_ = 3.219, *p* = 0.076).

### Effects of alpha-methyl-para-tyrosine

**Figure 3:**
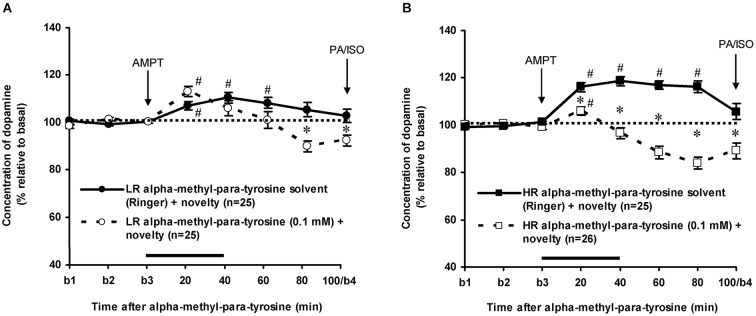
**Effects of intra-accumbens infusion of AMPT (0.1 mM, 2 μl/min, 40 min) on the DA levels in novelty-challenged low responders (LR) (A) and novelty-challenged high responders (HR) (B)**. Values are accumbal DA levels expressed as percentage of the average of the three baseline samples collected before AMPT was infused (basal samples b1-b3). Both PA and ISO were infused 100 min after AMPT was given. This infusion point was labeled basal sample b4. It is important to note that the DA levels of the control animals were returned to baseline when PA and ISO were infused, whereas the DA levels in AMPT-treated animals were still reduced. Data are expressed as mean percentage ± SEM. The solid horizontal line represents the infusion time of AMPT. ^#^ Significant DA increase after novelty (DA levels were compared to baseline levels using one sample *t*-tests). * Significant DA decrease after AMPT (DA levels of AMPT-treated rats were compared to DA levels of control (Ringer) animals using Student’s *t*-tests).

As previously reported (Saigusa et al., 1999; Verheij and Cools, 2007; Verheij et al., 2008), accumbal DA levels increased less in novelty-challenged LR than in novelty-challenged HR (Figure 3: rat type × time effect: *F*_(7,336)_ = 4.304, *p* < 0.001). The novelty-induced increase of DA lasted 60 min in LR (Figure 3A: one sample *t*-test: *t* = 20–60 min: *p* < 0.05 and *t* = 80–100 min: ns) and 80 min in HR (Figure 3B: one sample *t*-test: *t* = 20–80 min: *p* < 0.05 and *t* = 100 min: ns). AMPT strongly decreased the extracellular levels of accumbal DA in these rats (Figure 3: treatment × time effect (*t* = 0–100 min): *F*_(7,679)_ = 33.437, *p* < 0.001, rat type × treatment × time effect: *F*_(7,679)_ = 6.705, *p* < 0.001). AMPT did not reduce the increase of accumbal DA in the 60-min-lasting-period that DA increased in novelty-challenged LR (Figure 3A: Student’s *t*-test: *t* = 20–60 min: ns), whereas it reduced the DA increase during this period in novelty-challenged HR (Figure 3B: Student’s *t*-test: *t* = 20–60 min: *p* < 0.05). These results confirm our previous findings that the relative large novelty-induced increase of accumbal DA in HR is derived from AMPT-sensitive pools, whereas the relative small novelty-induced increase of accumbal DA in LR is derived from AMPT-resistant pools (Saigusa et al., 1999; Verheij and Cools, 2007). For the present study, it is important to note that AMPT decreased accumbal DA levels at 100 min after AMPT in both LR and HR rats (Figure 3: Student’s *t*-test: *t* = 100 min: LR: *p* < 0.05 and HR: *p* < 0.05; rat type × treatment effect (*t* = 100 min): ns).

### Rats are at rest during the infusion of isoproterenol and phentolamine

**Figure 4:**
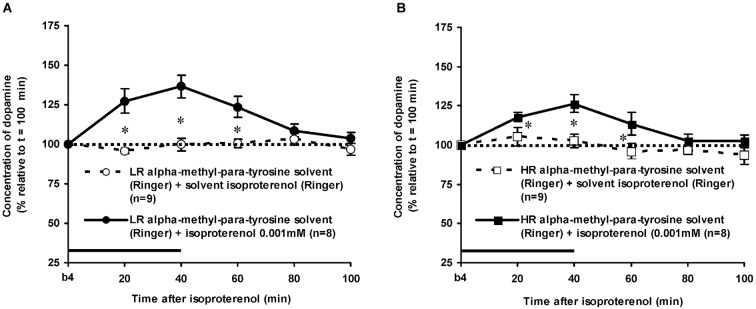
**Effects of the intra-accumbens infusion of the beta-adrenoceptor-agonist ISO (0.001 mM, 2 μl/min, 40 min) on the extracellular concentration of DA in the nucleus accumbens of LR (A) and HR (B) under non-novelty-challenged conditions**. All rats were treated with the solvent of AMPT. Values are accumbal DA levels expressed as percentage of the sample collected just before ISO was infused (basal sample b4 of Figure 3). Data are expressed as mean percentage ± SEM. The solid horizontal line represents the infusion time of ISO. * Significant DA increase after ISO (Student’s *t*-test).

As mentioned above, the DA levels of the control rats did not differ anymore from the baseline levels of DA at *t* = 100 min after novelty (Figure 3: one sample *t*-test at *t* = 100 min: LR: ns, HR: ns). In addition, the accumbal DA levels of these rats did not change over time during the period after *t* = 100 min (Figures 4, 6: time effect: LR: ns, HR: ns). These data demonstrate that both LR and HR were not anymore challenged at the time of the infusion of ISO or PA.

**Figure 5:**
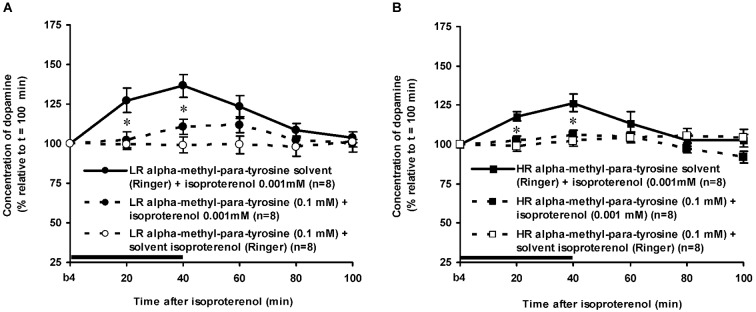
**Effects of AMPT (0.1 mM, 2 μl/min, 40 min) on the ISO-induced increase of the extracellular concentration of DA in the nucleus accumbens of non-challenged LR (A) and non-challenged HR (B)**. Values are accumbal DA levels expressed as percentage of the sample collected just before ISO was infused (basal sample b4 of Figure 3). Data are expressed as mean percentage ± SEM. The solid horizontal line represents the infusion time of ISO. * AMPT-induced reduction of the ISO-induced DA increase (Student’s *t*- test).

**Figure 6:**
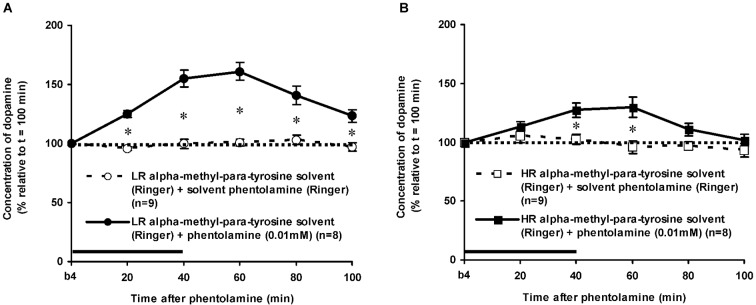
**Effects of the intra-accumbens infusion of the alpha-adrenoceptor-antagonist PA (0.01 mM, 2 μl/min, 40 min) on the extracellular concentration of DA in the nucleus accumbens of LR (A) and HR (B) under non-novelty-challenged conditions**. All rats were treated with the solvent of AMPT. Values are accumbal DA levels expressed as percentage of the sample collected just before PA was infused (basal sample b4 of Figure 3). Data are expressed as mean percentage ± SEM. The solid horizontal line represents the infusion time of PA. * Significant DA increase after PA (Student’s *t*-test).

### Effects of isoproterenol in alpha-methyl-para-tyrosine-treated rats

The dose of 0.001 mM of the beta-adrenoceptor-agonist ISO equally increased accumbal DA levels in non-challenged LR and non-challenged HR that were treated with the solvent of AMPT (Figure 4: treatment × time effect: *F*_(5,150)_ = 12.477, *p* < 0.001; rat type × treatment (× time) effect: ns). AMPT equally reduced this ISO-induced DA increase in both type of rats (Figure 5: treatment × time effect: *F*_(5,140)_ = 9.045, *p* < 0.001; rat type × treatment (× time) effect: ns). ISO did not increase anymore accumbal DA levels in either AMPT-treated LR or AMPT-treated HR (Figure 5: treatment (× time) effect: ns).

### Effects of phentolamine in alpha-methyl-para-tyrosine-treated rats

**Figure 7:**
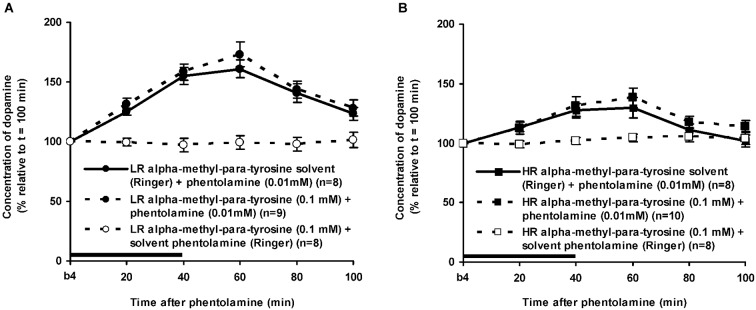
**Effects of AMPT (0.1 mM, 2 μl/min, 40 min) on the PA-induced increase of the extracellular concentration of DA in the nucleus accumbens of the non-challenged LR (A) and non-challenged HR (B)**. Values are accumbal DA levels expressed as percentage of the sample collected just before PA was infused (basal sample b4 of Figure 3). Data are expressed as mean percentage ± SEM. The solid horizontal line represents the infusion time of PA. There was no AMPT-induced reduction of the PA-induced DA increase (Student’s *t*-test).

The dose of 0.01 mM of the alpha-adrenoceptor-antagonist PA increased accumbal DA levels in both LR and HR that were treated with the solvent of AMPT (Figure 6). The PA-induced increase of accumbal DA was larger in non-challenged LR than in non-challenged HR (Figure 6: rat type × treatment × time effect: *F*_(5,150)_ = 8.911, *p* < 0.006; LR (Figure 6A): treatment × time effect: *F*_(5,75)_ = 21.436, *p* < 0.001; HR (Figure 6B): treatment × time effect: *F*_(5,75)_ = 4.929, *p* = 0.001). As in AMPT solvent-treated rats, PA increased accumbal DA levels more strongly in AMPT-treated LR than in AMPT-treated HR (Figure 7: rat type × treatment × time effect: *F*_(5,155)_ = 3.474, *p* = 0.005; LR (Figure 7A): treatment × time effect: *F*_(5,75)_ = 20.643, *p* < 0.001; HR (Figure 7B): treatment × time effect: *F*_(5,80)_ = 4.842, *p* = 0.001). In fact, the DA-increasing effects of PA in AMPT-treated rats were not different from the DA-increasing effects of PA in rats that were treated with AMPT solvent (Figure 7: treatment (× time) effect: ns).

## Discussion

We have previously demonstrated that binding of the beta-adrenoceptor agonist ISO to the postsynaptic beta-adrenoceptors of the nucleus accumbens increases accumbal DA release (Figure 1). The first aim of the present study was to investigate whether this ISO-induced DA release is derived from AMPT-sensitive pools of DA (see also Figure 1). In addition to the presynaptic acting alpha-adrenoceptor-agonist PE, the postsynaptic acting alpha-adrenoceptor-antagonist PA was also found to increase accumbal DA release (Figure 1). The second aim of this study was to investigate whether the DA-increasing effects of PA were like the DA-increasing effects of PE resistant to AMPT (see also Figure 1).

### Dual role of noradrenaline in mediating dopamine release

As expected, intra-accumbens infusion of PA and ISO increased accumbal DA levels in rats treated with the solvent of AMPT (Figures 4, 6). This accumbal DA increase may very well explain the previously reported increase in locomotor activity observed following the inhibition and stimulation of accumbal alpha- and beta-adrenoceptors, respectively (Verheij et al., accepted). It has been demonstrated previously that the DA increase following intra-accumbens administration of PA and ISO are dose-dependent and receptor-specific (Tuinstra and Cools, 2000). The present study also confirms the previously reported finding that the effects of ISO do not differ between non-challenged LR and HR (Tuinstra and Cools, 2000), whereas the effects of PA are larger in the former than in the latter (Tuinstra and Cools, 2000). These results have previously been ascribed to individual differences in the noradrenergic activity at accumbal postsynaptic alpha-, but not beta-adrenoceptors in non-challenged LR and HR rats (for details: Tuinstra and Cools, 2000).

### Effects of alpha-methyl-para-tyrosine on the dopamine release after isoproterenol or phentolamine

The finding that AMPT completely prevented the ISO-induced increase of accumbal DA levels in both LR (Figure 5A) and HR (Figure 5B) shows that ISO increases DA release from AMPT-sensitive DA pools. Importantly, the local infusion of AMPT completely depleted the newly-synthesized pools of DA in both rat types (Figure 5). Despite the fact that the newly-synthesized pools of DA were shown to be completely empty (Figure 5), the local infusion of PA led to an increase of accumbal DA levels in both AMPT-treated LR (Figure 7A) and AMPT-treated HR (Figure 7B). In fact, the PA-induced DA release in these AMPT-treated rats did not differ from the PA-induced DA release in rats treated with AMPT-solvent (Figure 7). The finding that not only the small PA-induced DA increase in HR, but also the large PA-induced DA increase in LR is insensitive to AMPT (Figure 7) illustrates that the PA-mediated DA release is independent on AMPT-sensitive pools of DA even when the release of DA is high.

## Conclusions

**Figure 8:**
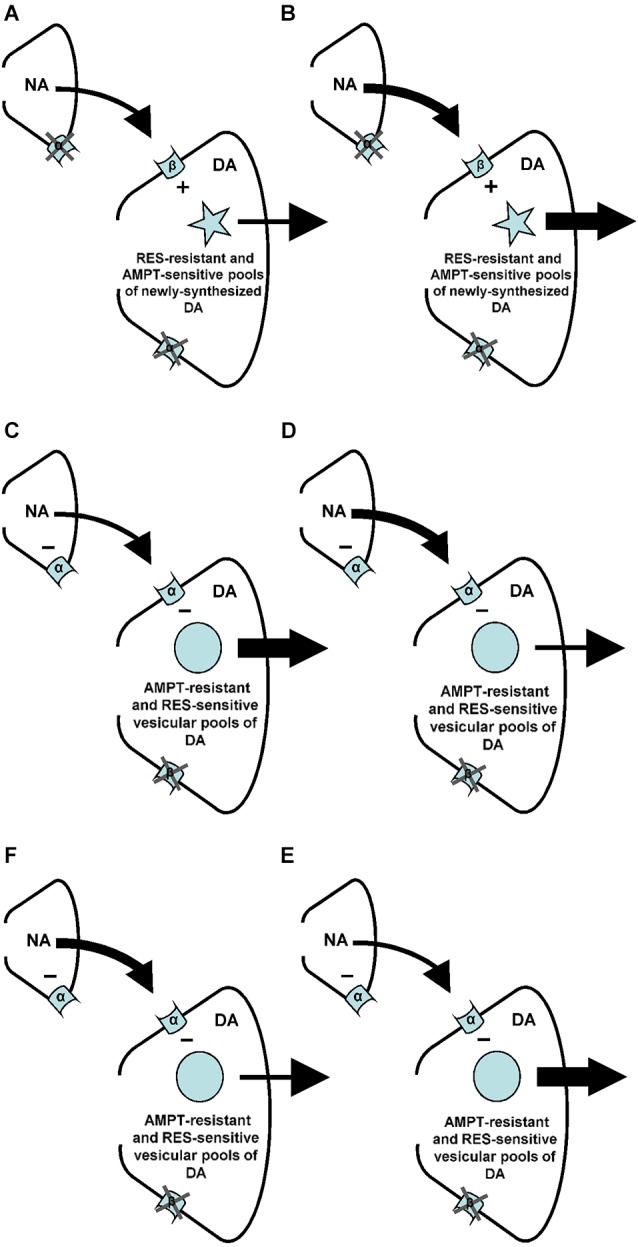
**Summary of the findings of the present and previous studies**. Changes in arrow weight represent changes in noradrenaline (NA) and/or DA release. **(A–B)** An increase of NA at the level of the postsynaptic beta (β)-, but not alpha (α)-adrenoceptors of the nucleus accumbens stimulates (+) accumbal DA release from AMPT-sensitive pools of newly-synthesized DA (present study) that are resistant to RES (see: Verheij and Cools, 2009b). **(C–D)** An increase of NA at the level of the postsynaptic alpha-, but not beta-adrenoceptors of the nucleus accumbens inhibits (–) accumbal DA release from RES sensitive vesicular DA pools (see: Verheij and Cools, 2009b) that are resistant to AMPT (present study). **(E–F)** The presynaptic alpha-adrenoceptors of the nucleus accumbens (see: Tuinstra and Cools, 2000; Verheij and Cools, 2009a) are known to inhibit (–) accumbal NA release (see: Aono et al., 2007). The present study indicates that this reduction of accumbal NA leads to an increase of accumbal DA (see also: Tuinstra and Cools, 2000; Verheij and Cools, 2009a) because the noradrenergic neurons containing presynaptic alpha-adrenoceptors impinge upon dopaminergic neurons equipped with postsynaptic alpha-, but not beta-receptors.

It has recently been shown that the increase of accumbal DA induced by ISO is due to the simultaneous stimulation of accumbal beta-1 and beta-2 receptors (Aono et al., 2013). The present finding that the ISO-induced increase of accumbal DA is sensitive to AMPT (see above) shows that the postsynaptic beta1/2-adrenoceptors of the nucleus accumbens mediate (i.e., stimulate) accumbal DA release from newly-synthesized pools of DA (Figure 8). In a previous study we have shown that the ISO-induced increase of accumbal DA release is not sensitive to reserpine (RES; Verheij and Cools, 2009b). This indicates that the AMPT-sensitive and newly-synthesized DA pools that are controlled by postsynaptic beta1/2-adrenoceptors are not vesicular (Figure 8). The PA-induced increase of accumbal DA is due to inhibition of alpha-1 but not alpha-2 receptors (Ihalainen et al., 2004; Saigusa et al., 2012). In contrast to the ISO-induced increase of accumbal DA, the PA-induced increase of accumbal DA was found to be sensitive to RES (Verheij and Cools, 2009b) demonstrating that the postsynaptic alpha-1-adrenoceptors of the nucleus accumbens mediate (i.e., inhibit) accumbal DA release from vesicular pools of DA (Figure 8). The present finding that the PA-induced increase of accumbal DA release is not sensitive to AMPT (see above) indicates that the RES-sensitive and vesicular DA pools controlled by alpha-1-adrenoceptors do not contain newly synthesized DA (Figure 8). The fact that the DA increase mediated by postsynaptic beta-1/2-adrenoceptors is sensitive to AMPT (present study), whereas the DA increase mediated by presynaptic alpha-1-adrenoceptors is not (Verheij and Cools, 2009a), indicates that the presynaptic alpha-1-adrenoceptors of the nucleus accumbens are not located on the noradrenergic nerve-terminals that impinge upon dopaminergic neurons expressing postsynaptic beta-1/2-receptors (Figure 8). The finding that both the DA increase mediated by presynaptic alpha-1-adrenoceptors (Verheij and Cools, 2009a) and the DA increase mediated by postsynaptic alpha-1-adrenoceptors (present study) are insensitive to AMPT confirms our hypothesis (see Section Introduction) that presynaptic alpha-1-adrenoceptors in the nucleus accumbens are located on those noradrenergic nerve-terminals that impinge upon dopaminergic neurons equipped with post-synaptic alpha-1-receptors (Figure 8).

Our study underlines the view that mesolimbic noradrenaline fulfills many functions that are, up to now, primarily ascribed to mesolimbic DA (Cools and Tuinstra, 2003). Indeed, both accumbal DA and accumbal noradrenaline were found not only to regulate locomotor activity (Pijnenburg et al., 1975; Costall et al., 1976; Cools et al., 1987; Ikeda et al., 2007; Verheij et al., accepted), sensorimotor gating (Alsene et al., 2010, 2011) and anxiety (Roozendaal and Cools, 1994; Kochenborger et al., 2012), but also more complex behaviors that are depending on learning and memory (Tuinstra et al., 2000, 2002; Kerfoot et al., 2008; Kerfoot and Williams, 2011) processes (for review: Verheij and Cools, 2008).

### Impact

Changes in accumbal DA are generally accepted to play a key role in addiction (Koob, 1992; Everitt and Robbins, 2005), schizophrenia (Gray et al., 1991; Gray, 1995; Carlsson et al., 2001; Kuepper et al., 2012) and Parkinson’s disease (Hornykiewicz, 1998; van Oosten et al., 2005). This explains why dopaminergic manipulations are widely used to treat these disorders. However, serious side effects of drugs that directly act at the dopaminergic receptors of the brain have been reported (Platt et al., 2002; Serretti et al., 2004; Ahlskog, 2011; Stowe et al., 2011; Meltzer, 2013). Partial DA agonists, however, are predicted to lead to fewer side effects because these drugs do not result in maximum stimulation of DA receptors (Jenner, 2002; Platt et al., 2002; Ohlsen and Pilowsky, 2005). Growing evidence indicates that moderate changes of endogenous DA levels in only a limited number of dopaminergic neurons, or at a limited number of dopaminergic receptors, may represent very powerful tools to treat dopaminergic diseases without producing severe side effects (Withers et al., 1995; Dixon et al., 1999; Müller, 2001; Mailman and Murthy, 2010; Diana, 2011). The fact that alpha- and beta-adrenoceptors control the release of DA from different types of dopaminergic pools (Figure 8, see also: Verheij and Cools, 2008, 2009b), which are in turn believed to control their own class of dopaminergic receptors (for review: Verheij and Cools, 2008), indicates that by choosing the appropriate noradrenergic treatment it may very well be possible to change the release of endogenous DA only from those pools that are involved in the therapeutic effects of DA, but not from the pools that induce DA-related side effects.

Whether an alpha- or beta-adrenergic agent has therapeutic potential depends on the disease to be treated. We have previously hypothesized that alpha-adrenoceptor-induced changes in vesicular DA release may be beneficial in the treatment of cocaine addiction and Parkinson’s disease, whereas beta-adrenoceptor-induced changes in the release of newly-synthesized DA may be beneficial in the treatment of amphetamine addiction and the positive symptoms of schizophrenia (for references see: Verheij and Cools, 2008). Indeed, it has recently been found that alpha-adrenoceptor antagonists reduce both the behavioral and accumbal DA response to cocaine (Mitrano et al., 2012), presumably by acting on the pre-synaptic alpha-1-adreneceptors of the nucleus accumbens (Verheij et al., 2013). In the future, additional animal studies will be needed to further investigate the beneficial effects of noradrenergic drugs as treatment for DA-related disorders.

## Conflict of interest statement

The authors declare that the research was conducted in the absence of any commercial or financial relationships that could be construed as a potential conflict of interest.

## Abbreviations

AMPT: alpha-methyl-para-tyrosine
DA: dopamine
HR: high responder(s) to novelty
ISO: isoproterenol
LR: low responder(s) to novelty
PA: phentolamine
PE: phenylephrine
RES: reserpine.
